# Dimesulfazet (Pesticides)

**DOI:** 10.14252/foodsafetyfscj.D-23-00007

**Published:** 2023-06-23

**Authors:** Food Safety Commission of Japan

## Abstract

Food Safety Commission of Japan (FSCJ) conducted a risk assessment of a sulfonanilide herbicide, dimesulfazet (CAS No. 1215111-77-5), based on results from various studies. The data used in the assessment include the fate in plants (paddy rice), residues in crops, fate in animals (rats), subacute toxicity (rats, mice and dogs), chronic toxicity (dogs), combined chronic toxicity/carcinogenicity (rats), carcinogenicity (mice), acute neurotoxicity (rats), subacute neurotoxicity (rats), two-generation reproductive toxicity (rats), developmental toxicity (rats and rabbits), and genotoxicity. The major adverse effects of dimesulfazet from those test results were observed in body weight (suppressed body weight gain in all test results), kidneys (increased weight in rats) and urinary bladder (urothelial hyperplasia in mice and dogs). None of carcinogenicity, neurotoxicity and genotoxicity were observed. No obvious effects on fertility were detected. The lowest no-observed-adverse-effect level (NOAEL) obtained from all the studies was 0.39 mg/kg bw per day in two-year combined chronic toxicity/carcinogenicity study in rats. On the basis of this value, FSCJ specified an acceptable daily intake (ADI) of 0.0039 mg/kg bw per day after applying a safety factor of 100 to the NOAEL. The lowest NOAEL for potential adverse effects after a single oral administration of dimesulfazet was 15 mg/kg bw per day in the developmental toxicity study in rabbits. FSCJ thus specified an acute reference dose (ARfD) of 0.15 mg/kg bw after applying a safety factor of 100 for women who are pregnant or might be pregnant. For the general population, an ARfD of 0.41 mg/kg bw after applying a safety factor of 300 (additional factor 3 by applying LOAEL of 125 mg/kg bw resulted from acute neurotoxicity study in rats).

## Conclusion in Brief

Food Safety Commission of Japan (FSCJ) conducted a risk assessment of a sulfonanilide herbicide, dimesulfazet (CAS No. 1215111-77-5), based on results from various studies.

The data used in the assessment include the fate in plants (paddy rice), residues in crops, fate in animals (rats), subacute toxicity (rats, mice and dogs), chronic toxicity (dogs), combined chronic toxicity/carcinogenicity (rats), carcinogenicity (mice), acute neurotoxicity (rats), subacute neurotoxicity (rats), two-generation reproductive toxicity (rats), developmental toxicity (rats and rabbits), and genotoxicity.

The major adverse effects of dimesulfazet from those test results were observed in body weight (suppressed body weight gain in all test results), kidneys (increased weight in rats) and urinary bladder (urothelial hyperplasia in mice and dogs). None of carcinogenicity, neurotoxicity and genotoxicity were observed. No obvious effects on fertility were detected.

In developmental toxicity study in rabbits, an increase in the number of dams having abnormal fetuses and an increase in the number of abnormal fetuses (cardiovascular abnormalities) were observed.

Based on the results from various studies, dimesulfazet (parent compound only) was identified as the relevant substance for the residue definition for dietary risk assessment in agricultural products, fish and shellfish.

The lowest no-observed-adverse-effect level (NOAEL) obtained from all the studies was 0.39 mg/kg bw per day in two-year combined chronic toxicity/carcinogenicity study in rats. On the basis of this value, FSCJ specified an acceptable daily intake (ADI) of 0.0039 mg/kg bw per day after applying a safety factor of 100 to the NOAEL.

The lowest NOAEL for potential adverse effects after a single oral administration of dimesulfazet was 15 mg/kg bw per day in the developmental toxicity study in rabbits. The findings were an increased number of dams having abnormal fetuses and an increased number of abnormal fetuses (cardiovascular abnormalities). FSCJ thus specified an acute reference dose (ARfD) of 0.15 mg/kg bw after applying a safety factor of 100 for women who are pregnant or might be pregnant. For the general population, an ARfD of 0.41 mg/kg bw after applying a safety factor of 300 (additional factor 3 by applying LOAEL of 125 mg/kg bw resulted from acute neurotoxicity study in rats). 

**Table 1.** *Levels relevant to toxicological evaluation of dimesulfazet*

Species	Study	Dose (mg/kg bw per day)	NOAEL (mg/kg bw per day)	LOAEL (mg/kg bw per day)	Critical endpoints^1^^)^
Rat	90-day subacute toxicity study	0, 30, 100, 300, 1 000 ppm	M: 2.2 F: 10.5	M: 7.9 F: 29.8	M/F: Suppressed body weight gain, etc.
		M: 0, 2.2, 7.9, 24.3, 78.8 F: 0, 2.9, 10.5, 29.8, 115			
	Two-year combined chronic toxicity /carcinogenicity study	0, 10, 30, 150, 300 ppm	M: 0.39 F: 1.53	M: 1.18 F: 7.71	M: Increase of relative kidney weight and increase of BUN F: Suppressed body weight gain, etc.
		Carcinogenicity group M: 0, 0.39, 1.18, 5.87, 11.9 F: 0, 0.49, 1.53, 7.71, 15.7 52-week emergently slaughtered group M: 0, 0.47, 1.43, 7.18, 14.1 F: 0, 0.59, 1.76, 8.86, 18.7			
	90-day subacute neurotoxicity study	0, 30, 100, 300 ppm	M: 6.93 F: 28.2	M: 21.0 F: -	M: Suppressed body weight gain and decreased feed consumption F: No toxicity
		M: 0, 2.12, 6.93, 21.0 F: 0, 2.77, 9.26, 28.2			
	Two-generation reproductive toxicity study	0, 60, 200, 600 ppm	Parent PM: 3.8 PF: 4.7 F_1_M: 5.2 F_1_F: 5.4 Offspring PM: 12.8 PF: 15.6 F_1_M: 17.3 F_1_F: 17.8	Parent PM: 12.8 PF: 15.6 F_1_M: 17.3 F_1_F: 17.8 Offspring PM: 38.8 PF: 46.7 F_1_M: 52.6 F_1_F: 53.8	Parent M/F: Suppressed body weight gain, etc. Offspring M/F: Suppressed body weight gain (No effect on fertility is observed)
		Parent generation PM: 0, 3.8, 12.8, 38.8 PF: 0, 4.7, 15.6, 46.7 Offspring generation F_1_M: 0, 5.2, 17.3, 52.6 F_1_F: 0, 5.4, 17.8, 53.8			
	Developmental toxicity study	0, 5, 15, 50	Dams: 50 Fetuses: 15	Dams: - Fetuses: 50	Dams: No toxicity Fetuses: Low body weight (No teratogenicity is observed)
Mouse	90-day subacute toxicity study	0, 100, 300, 1 000 ppm	M: 37.1 F: 17.2	M: 126 F: 47.8	M: Suppressed body weight gain, etc. F: Decrease of TP
		M: 0, 12.8, 37.1, 126 F: 0, 17.2, 47.8, 166			
	18-month carcinogenicity study	0, 20, 60, 200, 600 ppm	M: 2.04 F: 2.42	M: 6.40 F: 7.12	M/F: Increase of pigmented (lipofuscin) macrophage of the cecum
		M: 0, 2.04, 6.40, 20.7, 62.8 F: 0, 2.42, 7.12, 25.7, 75.7			
Rabbit	Developmental toxicity study	0, 5, 15, 50	Dams: 15 Fetuses: 15	Dams: 50 Fetuses: 50	Dams: Decreased in defecation and decreased body weight Fetuses: Increased number of dams with abnormal fetuses and increased number of abnormal fetuses (with cardiovascular abnormalities)
Dog	90-day subacute toxicity study	0, 3, 10, 30	M: 10 F: 3	M: 30 F: 10	M: Suppressed body weight gain, etc. F: Increase of BUN
	One-year chronic toxicity study	0, 1.5, 5, 15	M/F: 5	M/F: 15	M: Increase of BUN F: Suppressed body weight gain, etc.
ADI			NOAEL: 0.39 SF: 100 ADI: 0.0039		
The critical study for setting ADI			Two-year combined chronic toxicity/carcinogenicity study in rat		

ADI, Acceptable daily intake; NOAEL, No-observed-adverse-effect level; LOAEL, Lowest-observed-adverse-effect level; SF, Safety factor; BUN, Blood urea nitrogen; TP, Total protein

-, NOAEL nor LOAEL could not be specified.

^1)^ The adverse effect observed at LOAEL.

**Table 2.** 1 *Potential adverse effects of a single oral administration of dimesulfazet* (*General population*)

Species	Study	Dose (mg/kg bw)	Endpoints relevant to setting NOAEL and ARfD (mg/kg bw)^1^^)^
Rat	Acute toxicity study	F: 300, 2 000	F: - F: Hunchback position
	Acute neurotoxicity study	M/F: 0, 125, 250, 500	M: - F: 125 M: Decreased in locomotor activity F: Decrease in activities, decreased times of standing upright and decreased in locomotor activity
ARfD			LOAEL: 125 SF: 300 ARfD: 0.41
The critical study for setting ARfD			Acute neurotoxicity study in rat

ARfD, Acute reference dose; LOAEL, Lowest-observed-adverse-effect level; SF, Safety factor; -, NOAEL could not be specified.

^1)^ The adverse effect observed at LOAEL.

**Table 2.** 2 *Potential adverse effects of a single oral administration of dimesulfazet* (*women who are pregnant or might be pregnant*)

Species	Study	Dose (mg/kg bw per day)	Endpoints relevant to setting NOAEL and ARfD (mg/kg bw per day)^1^^)^
Rabbit	Developmental toxicity study	0, 5, 15, 50	Fetuses: 15 Fetuses: Increased number of dams with abnormal fetuses and increased number of abnormal fetuses (with cardiovascular abnormalities)
ARfD			NOAEL: 15 SF: 100 ARfD: 0.15
The critical study for setting ARfD			Developmental toxicity study in rabbit

ARfD, Acute reference dose; NOAEL, No-observed-adverse-effect level; SF, Safety factor

^1)^ The adverse effect observed at LOAEL.
